# An intersectional approach to examine low birthweight by migration status in Sweden using register data

**DOI:** 10.1038/s41598-025-26172-0

**Published:** 2025-11-04

**Authors:** Sooz Romero, Andrea Dunlavy, Lisa Berg, Sol P. Juárez

**Affiliations:** 1 Department of Public Health Sciences, Stockholm University, Stockholm, Sweden; 2 Centre for Health Equity Studies (CHESS), Stockholm University/Karolinska Institutet, Stockholm, Sweden

**Keywords:** Inequalities, International migrants, Perinatal health, Birth outcomes, Intersectionality, Risk factors, Socioeconomic scenarios

## Abstract

This study aimed to compare differences in low birthweight (LBW, < 2500 g) between the offspring of Swedish-born women and migrant women from the Global North and Global South, while simultaneously considering the role of other social factors. We used total population registers to study resident women who gave birth in Sweden between 1997 and 2016, representing a total of 1,917,171 live births. Logistic regression analyses were performed using two analytical approaches: (1) a standard approach, applying regression models to assess the association between region of birth and LBW while adjusting for social factors; and (2) an intersectional approach, with an additive combination of region of birth, income, and parental support variables. Both approaches yielded odds ratios (OR) with 95% confidence intervals (CI) and were adjusted for year of birth, maternal age, and parity. Results from both approaches showed differences in the odds of LBW according to maternal background. Importantly, findings from the intersectional approach demonstrated that women from the Global South had consistently higher odds of LBW across income levels, but with risks amplified among those living alone. These findings show that maternal migration background is a strong determinant of LBW, and that its impact is differentially compounded by social factors.

**Supplementary Information:**

The online version contains supplementary material available at 10.1038/s41598-025-26172-0.

## Introduction

Low birthweight (LBW) is a child health indicator associated with increased risk of mortality and morbidity in later life^1,2^. Social inequalities are a major determinant of LBW, thus making this health outcome an important factor in the perpetuation of inequalities across generations. Migration background, along with gender, class, race and ethnicity, is a recognized dimension of social stratification, which can act as an axis of inequality in society^3^. Despite this, migrant women do not uniformly experience higher risks of giving birth to LBW babies^4^. This evidence is consistent with the general phenomenon of the ‘healthy migrant paradox’ which describes the observation that migrants often exhibit an unexpected health advantage compared to the native-born majority population, despite facing poorer socioeconomic conditions in the new context^5^.

The presence of the migrant health advantage has contributed to the isolation of migration research from studies of other forms of social inequality. Yet the extent to which the migrant health advantage can be considered a universal phenomenon has increasingly been questioned, due to variations across outcomes^6^, health dimensions^7^ and over time (both historical and over the life course)^8^, as well as variations by migrant origin- and receiving-country contexts^9^. In Sweden for example, although adult migrants (both men and women) have a mortality advantage relative to the native-born majority population^7^, their children have a higher risk of being born as LBW babies than do children of Swedish-born mothers^10^, suggesting increasing inequalities across migrant generations, particularly among those with non-Western backgrounds^10^.

Intersectionality theory, originally formulated in relation to experiences of gender and racial inequality^11^, provides a relevant perspective for examining migrant health. The theory recognizes that individuals are situated within multiple and interlocking systems of inequality^11^. Within these complex intersections, health risks are shaped in unique ways, highlighting the limitations of focusing solely on one form of social stratification (such as migration) over others (such as social class). Thus, an intersectional lens can help to reposition evidence on migration and health within the framework of social inequality research^12^.

In this paper, we adopt an intersectional lens within the context of global public health research^13^ to examine perinatal health outcomes (specifically LBW) among the offspring of migrant women in Sweden coming from the Global North and the Global South (the so-called ‘Brandt line’ categorization)^14^. By moving away from the traditional categorization of migrant origin based on geographical proximity, we aim to make visible a particular form of power dynamics that are embedded in global inequalities. Such inequalities manifest in the characterization of a ‘poor South’ as a source of cheap labour and raw materials for an economic and political influential ‘rich North’. These power dynamics precede migration, yet can shape migrants’ experiences in Sweden in terms of, for example, restricted access to visas, precarious legal status, racism and discrimination, and limited opportunities for upward social mobility.

This study further examines the Global North–South divide by simultaneously considering how other social factors may be differentially associated with health among migrant mothers relative to the Swedish-born population. Specifically, we examine the role that income and partner support (proxied through cohabitation) may have for the low birthweight of offspring born to Global North and Global South migrant women. Income and partner support may affect migrants and natives in different ways. In a context such as Sweden, where universal access to health care is provided to all registered residents on equal terms, income may still play a significant role as a determinant of health care utilization. For instance, individuals with greater financial resources may choose to pay for private care to reduce waiting times and access potentially higher-quality healthcare services. Similarly, wealthier labour migrants who find it difficult to navigate the public healthcare system may consider paying for private care to overcome these barriers. This can create new forms of health inequality prior to pregnancy, both between migrant groups with different income levels and relative to the Swedish-born population, whereby individuals with higher incomes may secure advantages in healthcare access and quality, despite the principle of universality. Similarly, while partner support is generally important for all expectant mothers^15^, the negative effects of lone motherhood on child health may be more pronounced for women with reduced family networks, which tends to be more likely among migrant women^16^. Therefore, standard approaches which assess the risk of LBW between the offspring of migrants and natives by ‘controlling’ for various social factors may not be sufficient. An approach that examines differences in the risk of LBW between the offspring of migrants and Swedish-born mothers while simultaneously acknowledging the potential distinct effects of social factors in each group is needed.

Inspired by intersectionality theory, we aim to advance knowledge on migration background and perinatal health by comparing LBW in the offspring of Swedish-born women and migrant women from the Global North and the Global South, while simultaneously considering how factors such as income and partner support (i.e., including both material and psychosocial aspects) may differentially affect these groups.

## Methods

### Data sources and study population

Information from several linked Swedish population registers was used for this study. This included the Medical Birth Register (MBR), which covers 98% of all births in Sweden and includes information on birthweight, gestational age, year of birth, and maternal age. From the Longitudinal Integrated Database for Health Insurance and Labour Market Studies (LISA), we retrieved socio-demographic information on disposable income, civil status, and cohabitation. From the Total Population Register (TPR) we retrieved information on maternal country/region of birth. Pseudonymized identification numbers were used to link individuals across the registers, ensuring anonymity^17^.

**Fig. 1:**
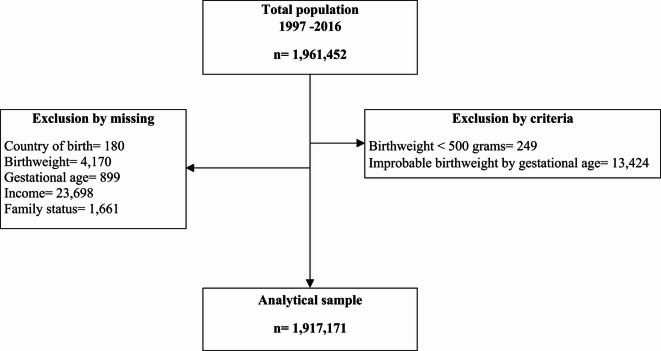
Flowchart depicting exclusions and final analytical sample.

The study population was comprised of all singleton live births to women who gave birth in Sweden from 1997 to 2016 in hospitals (n = 1,961,452 births). We excluded observations of newborns with recorded birthweights of less than 500 g (n = 249), or with improbable combinations of birthweight by gestational week (n = 13,424)^18^, and missing information on either gestational age (n = 899), birthweight (n = 4170), mother’s country of birth (n = 180), disposable income (n = 23,698), and cohabitation (n = 1661), leaving an analytical sample of 1,917,171 observations **(**Fig. 1**).**

### Low birthweight

Our outcome of interest was LBW defined as a birthweight less than 2500 g, regardless of gestational age^19^.

### Region of birth

Region of birth was categorized into three groups: Sweden, Global North and Global South. The Global North included European and other high-income countries, such as North America. The Global South included low- and middle-income countries from Asia, Africa, South America, and Europe (excepting EU28 and Nordic countries). The Global North/South categorization is increasingly proposed in migration and health research to emphasise the role of global power inequalities over categorizations that emphasize geographical, ethnic or cultural dimensions. Although there is not a common agreed-upon definition of countries, we present the detailed list of the countries we used in a Supplementary note.

### Income and partner support

Income was measured as disposable individualized income (defined as total income received, including allowances, minus taxes) from the year before the birth of the child^20^. This variable was divided into tertiles: low income, middle income, and high income. Partner support was proxied by information on household composition in the year prior to the child’s birth, categorized as cohabitating or living alone. Cohabitation included mothers who were married or living with the other parent. Living alone included mothers who were single or who were living without the other parent.

### Covariates

Additional covariates included: year of birth, used to account for potential calendar effects and divided into: 1997–2001, 2002–2006, 2007–2011 and 2012–2016; maternal age at delivery, divided into three age categories: < 24, 25–35, and > 35; parity, categorized into primiparous and multiparous, ^15^ and gestational age, categorized into preterm (< 37 weeks), term (37–41 weeks) and post- term (≥ 42 weeks).

### Statistical analysis

We employed two analytical approaches: a standard approach and an intersectional approach. For the standard approach we conducted logistic regression analyses to examine the association between region of birth and LBW, using Swedish-born women as the reference group. From this approach, odds ratios (OR) with 95% confidence intervals (CI) were derived from two different models. The first model included year of birth, maternal age and parity, and the second model was additionally adjusted for income and partner support.

For the intersectional approach we employed a method proposed by previous studies^21–25^, and created an intersectional variable as an additive combination of region of birth (categorized as Sweden, Global North or Global South), income (low, middle, or high) and partner support (cohabiting or living alone). This resulted in a variable with eighteen categories. In these analyses, the association between this intersectional exposure variable and LBW was examined, using Swedish-born cohabiting women with high income as the reference group. As in the standard approach, the analyses using the intersectional exposure variable were also adjusted for year of birth, maternal age and parity. We estimated robust standard errors by clustering the observations based on mother’s ID, thereby accounting for the presence of siblings within the study population.

All statistical analyses were conducted using the STATA software version SE 16.0 ^26^.

### Ethical approval

The ethical review board waived the necessity of informed consent due to the anonymized nature of the data material and the study has received approval from the Regional Ethical Review Board of Stockholm (decision no. 2017/716-31/5). The data were processed in accordance with the General Data Protection Regulation and all methods were performed in accordance with the relevant guidelines and regulations (Declaration of Helsinki).

## Results

### Population characteristics

Table 1 shows the characteristics of the study population by origin. Swedish-born women constituted 79.3% of the study population, followed by women from the Global South (16.0%) and women from the Global North (4.7%). The overall proportion of LBW was 2.7%, with the highest proportion observed among mothers from the Global South (3.4%). Low income was more prevalent in the Global South group (66.4%) compared to the native-born Swedish group (26.2%). The majority of the study population cohabitated (69.5%), with the highest proportion seen among mothers from the Global South (76.6%). The highest proportion of women living alone was observed among Swedish-born mothers (34.6%), while the Global South group had the lowest proportion (23.3%).

**Table 1:** Characteristics of the study population by region of birth (n = 1,917,171).

		**Total**	**Swedish-born**	**Global North**	**Global South**
		N = 1,917,171	N = 1,520,156	N = 91,424	N = 305,591
			(79.3%)	(4.8%)	(15.9%)
Birthweight in grams	< 2500	52,971 (2.7%)	40,114 (2.6%)	2,603 (2.8%)	10,254 (3.4%)
	2500–3999	1,486,385 (77.5%)	1,159,533 (76.3%)	72,230 (79%)	254,622 (83.3%)
	≥ 4000	377,815 (19.7%)	320,509 (21%)	16,591 (18.1%)	40,715 (13.3%)
Gestational weeks	< 37	68,054 (3.5%)	54,093 (3.6%)	3,149 (3.4%)	10,812 (3.5%)
	37–41	1,594,818 (83.2%)	1,260,720 (83%)	76,428 (83.54%)	257,670 (84.3%)
	≥ 42	245,299 (13.3%)	205,343 (13.5%)	11,847 (13%)	37,109 (12.1%)
Disposable Income	Low	639,013 (33.3%)	398,136 (26.2%)	37,280 (40.8%)	203,597 (66.6%)
	Medium	638,377 (33.3%)	550,560 (36.2%)	25,728 (28.1%)	62,089 (20.3%)
	High	639,781 (33.4%)	571,460 (37.6%)	28,416 (31%)	39,905 (13%)
Partner support	Cohabitating	1,293,811 (67.5%)	993,954 (65.4%)	65,486 (71.6%)	234,371(76.6%)
	Living alone	623,360 (32.5%)	526,202 (34.6%)	25,938 (28.3%)	71,220 (23.3%)
Mother’s age at delivery	< 24	271,862 (14.2%)	203,134 (13.4%)	8,830 (9.6%)	59,898 (19.6%)
	25–34	1,258,567 (65.6%)	1,016,822 (66.9%)	57,585 (63%)	184,160 (60.2%)
	≥ 35	386,742 (20.2%)	300,200 (19.7%)	25,009 (27.3%)	61,533 (20.1%)
Year of birth	1997–2001	408,382 (21.3%)	340,714 (22.4%)	18,507 (20.2%)	49,161 (16%)
	2002–2006	463,491 (24.1%)	381,05 (25%)	20,088 (22%)	62,353 (20.4%)
	2007–2011	512,248 (26.7%)	401,158 (26.4%)	24,961 (27.3%)	86,129 (28.1%)
	2012–2016	533,050 (27.8%)	397,234 (26.1%)	27,868 (30.5%)	107,948 (35.3%)
Parity	Primiparous #1	272,562 (14.2%)	210,035 (13.8%)	17,687 (19.3%)	44,840 (14.6%)
	Multiparous #2 +	1,644,609 (85.8%)	1,310,121 (86.1%)	73,737 (80.6%)	260,751 (85.3%)

## Standard and intersectional analytical approaches

Table 2 presents ORs from logistic regression models examining the association between origin and LBW using the standard approach, as well the association between the combination exposure variable and LBW using the intersectional approach. The standard approach analyses showed that, compared to Swedish-born mothers, increased odds of delivering a child with LBW were observed among mothers from the Global South (OR 1.34, 95% CI 1.31-1.38), and, to a lesser degree, among mothers from the Global North (OR 1.06, 95% CI 1.01-1.10).

**Table 2:** Multivariable regression analysis between low birthweight, partner support and income (n = 1,917,171).

	Low birthweight (n)	Standard approach		Intersectional approach
		Model 1		Model 2
		OR (CI. 95%)	OR (CI. 95%)	OR (CI. 95%)
Region of birth				
Sweden	40,114	Ref	Ref	
Global North	2603	1.03 (0.99–1.08)	1.06 (1.01–1.10)	
Global South	10,254	1.28 (1.25–1.31)	1.34 (1.31–1.38)	
Income				
High	18,667		Ref	
Medium	16,644		0.98 (0.96–1.01)	
Low	17,660		0.99 (0.97–1.01)	
Partner’s support				
Cohabiting	31,341		Ref	
Living alone	21,630		1.34 (1.31–1.37)	
Intersectional variable				
Swedish/Cohabiting/High income	6557			Ref
Swedish/Cohabiting/Medium Income	8545			0.96 (0.93–0.99)
Swedish/Cohabiting/Low Income	7143			1.00 (0.97–1.04)
Swedish/Living Alone/High Income	9704			1.36 (1.31–1.40)
Swedish/ Living Alone/Middle Income	5238			1.40 (1.35–1.46)
Swedish/ Living Alone/Low Income	2927			1.37 (1.31–1.43)
Global North/Cohabiting/High Income	459			1.07 (0.97–1.18)
Global North/Cohabiting/Middle Income	488			1.04 (0.94–1.15)
Global North/Cohabiting/Low Income	724			1.11 (1.02–1.20)
Global North/ Living Alone/High Income	369			1.34 (1.20–1.49)
Global North/ Living Alone/Middle Income	220			1.49 (1.29–1.71)
Global North/ Living Alone/Low Income	343			1.36 (1.21–1.52)
Global South/Cohabiting/High Income	841			1.48 (1.38–1.60)
Global South/Cohabiting/Middle Income	1391			1.40 (1.31–1.48)
Global South/Cohabiting/Low Income	5193			1.38 (1.33–1.43)
Global South/ Living Alone/High Income	737			1.79 (1.65–1.93)
Global South/ Living Alone/Middle Income	762			1.78 (1.65–1.92)
Global South/ Living Alone/Low Income	1330			1.53 (1.44–1.63)

All models adjusted for year of birth, maternal age, and parity

The intersectional approach analyses indicated that, compared to cohabiting Swedish-born mothers with high income, Swedish-born mothers living alone had higher odds of LBW, regardless of income level (ORs ranging from 1.36 to 1.40). Cohabitating mothers from the Global North had odds of LBW that were similar to the reference group, except for those with low income, who showed slightly elevated odds of LBW (OR 1.11, 95% CI 1.02-1.20). Compared to the reference group, mothers from the Global North living alone had higher odds of LBW, irrespective of income level (ORs ranging from 1.34 to 1.49), demonstrating odds which were comparable to those of Swedish-born mothers living alone. Mothers from the Global South had consistently elevated odds of LBW across all income levels, with associations being slightly more pronounced among mothers living alone (ORs ranging from 1.53 to 1.79) compared to cohabiting mothers (ORs ranging from 1.38 to 1.48).

## Discussion

This study examined inequalities in LBW between the offspring of Swedish-born and migrant mothers, while considering the possible differential roles of income and partner support. Our study showed that mothers who lived alone (without partner support) were more likely to give birth to LBW infants compared to cohabiting mothers, regardless of income level. This was observed across all maternal origins but was most pronounced among migrant mothers, particularly those from the Global South. Overall, our findings confirm the need to move beyond a simplistic dichotomy of migrant vs. non-migrant mothers when examining inequalities in birth outcomes, as the risks of LBW emerge at the intersection of migration background and other social factors, including income and partner support. Moreover, the study highlights the need for an intersectional approach in migration and health research, which considers differences within migrant groups that arise from a complex interplay of social factors that differentially affect these groups. At the same time, the observed differences across migrant maternal origins, regardless of income and cohabitation (with women from the Global South showing a higher risk of LBW than those from the Global North, and also higher risk than the majority Swedish population) indicate that migration background itself constitutes an independent form of inequality. This inequality should not be reduced to or explained away by other forms of disadvantage.

The role of partner support in influencing birth outcomes is well documented in the literature ^27^, reflecting both material and psychosocial mechanisms. The current study provides empirical support for previous research, which concludes that lone parents are an increasingly vulnerable group^36^. Earlier studies have shown that single or lone parents face elevated risks of poverty, particularly as welfare benefits have become less generous over time, including in the Nordic region^28^. In addition to financial constraints, partner support is particularly relevant for LBW, as pregnancy is a sensitive and potentially stressful period for women, involving physical, hormonal and emotional challenges that may be more difficult to manage without partner support^27,29,30^. In our study, the relevance of partner support is supported by the evidence that lone mothers tended to have higher risks of LBW across origins and income levels. However, it is also relevant to highlight that cohabitation plays a greater role among mothers from the Global South, followed by mothers from the Global North and the Swedish-born population.

Our findings indicate that income does not play a substantial role in explaining differences in LBW between the offspring of migrant and Swedish-born mothers. The relatively higher contribution of cohabitation compared to income levels in our study is a novel finding; however, it should be interpreted with caution. Previous studies have demonstrated income to be a key indicator of socioeconomic differences in birth outcomes^31^, and the quality of income information from the Swedish national registers is highly reliable^32^. However, women’s participation in the labour market is not independent from their reproductive experiences, which have implications for earned income. For example, Swedish mothers often take more and relatively longer periods of parental leave and are more likely to return to the labour market on a part-time basis compared to migrant women^33^. This may narrow the income gap between Swedish-born and migrant mothers during their reproductive years, potentially attenuating the role of income in adverse perinatal health outcomes. Although we measured disposable income in the year before birth to minimize such discrepancies, we cannot eliminate them entirely. Moreover, other dimensions of socioeconomic position, including education level, labour market attachment, occupational position, and quality of working and living conditions, are likely important for understanding socioeconomic differences in LBW between these groups and should be examined in future research. For instance, a recent study demonstrated higher odds of giving birth to a child who was small for gestational age, a birth outcome related to LBW, among women with low education^21^. Notably, the highest odds were observed among young, unmarried women with low education who were born outside of Sweden^21^, aligning with our findings on the significant role of social support for perinatal health outcomes. However, information on educational attainment is also not without specific limitations in the Swedish registers. Unlike income data, the educational attainment of migrants who have not completed their studies in Sweden is often based on self-reported information collected through mail surveys and other administrative data. This represents a source of uncertainty in the statistics on educational attainment among the foreign-born, as partial non-responses from surveys and other supplemental data sources are recorded as missing in the registers. For example, estimates from 2023 showed that missingness in the Education register was 9.3% among the foreign-born, compared to only 0.6% among the Swedish-born^34^.

The observed differences in the risk of LBW between migrants from the Global North and the Global South suggest that migration, indicative of global forms of inequality, plays an independent role in shaping the risk of LBW. These differences may reflect global inequalities existing prior to migration, but also, and perhaps more importantly, differences in the experiences of migrants in Sweden. The mortality advantage observed among migrants upon arrival^7^ (i.e., a summary measure of overall health at the population level) tends to disappear with increasing duration of residence^35^. The attenuation of this advantage, alongside evidence on the worse perinatal health outcomes of migrants’ descendants^10^, suggests that some of these inequalities may be rooted in post-migration experiences, with intergenerational effects. Specific forms of inequality that could impact the Global South group to a greater extent include discrimination and racism, which can be perpetuated across generations. Notably, a study conducted in Sweden found that inequalities in birthweight increase over generations following a Western/non-Western origin divide (an alternative categorization to the Global South and North), illustrating how patterns of racial inequality develop in the receiving country^10^.

Furthermore, the differences in the risk of LBW observed between mothers from the Global South and North across all combinations of income levels and cohabitation categories highlights the importance of adopting an intersectional approach to the study of perinatal health. In other words, our results show that the experience of having a low income and/or being a single mother differs depending on whether one is a Swedish-born mother, a migrant mother from the Global South or a migrant mother from the Global North. This finding can be interpreted in two ways that are not mutually exclusive: on the one hand, the relative differences in disadvantages between migrants from the Global North and Global South may result from a differential effect of income and cohabitation on LBW. On the other hand, coming from the Global North and Global South may put people at risk of a wide range of diverse disadvantages in Sweden beyond objectively similar disadvantages (income and cohabitation). Although it is beyond the scope of this study to determine which of these possibilities are at play, our findings clearly show that migrants are a heterogeneous group with unique yet diverse experiences of inequality. This heterogeneity further highlights the limitations of comparing perinatal health outcomes between migrants and the native-born using only socioeconomic adjustments, which assumes that these variables have the same effect across groups with differences in the levels of prevalence. This standard approach indirectly assumes that differences in the risk of LBW between these groups can ultimately be reduced to (“explained by”) identifying a set of observable factors. However, our findings, interpreted from an intersectional perspective, may also suggest that the inequalities involved may be intrinsic to the lived experience of being a migrant, particularly from the Global South. The differences in the risk of LBW observed in this study between the offspring of mothers from the Global South and Global North are a marker of other forms of social inequality, as LBW is associated with multiple health and social outcomes across the life course. Identifying and addressing inequalities in the receiving country that affect parents, especially mothers, from the Global South and Global North is therefore crucial to ensuring healthy lives and promoting well-being at all ages, in line with Sustainable Development Goal 3.

## Strengths and limitations

A major strength of our study is the use of Swedish national registers, which covered almost all births in Sweden during the study period, and are linked to social registers that provide accurate and representative information. Nevertheless, the study has limitations that should be considered. First, even though the use of Swedish national registers allowed us to investigate these associations in a total population setting, facilitating our application of an intersectional approach without encountering statistical power issues due to small sample sizes, there are also limitations of the registers. For example, cohabitation was used as a proxy for partner support, which does not fully capture living arrangements, as unmarried but cohabiting couples without joint children are not registered as cohabiting^32^. And, importantly, registry information does not allow us to consider the quality of relationships, as cohabitation or marriage does not always guarantee better social support, as studies on domestic violence and birth outcomes have demonstrated^19^. Finally, due to ethical reasons, the available data on country of birth is often aggregated by region^20^, particularly for countries with smaller populations resident in Sweden, in order to better ensure anonymity. Origin categorizations by region may entail a risk of misclassification, however this is likely a minor issue given the broader Global North and Global South categories that were employed.

## Concluding remarks

This study examined inequalities in LBW between the offspring of Swedish-born women and migrant women from the Global North and the Global South. Our findings demonstrated that women from the Global South had consistently higher odds of LBW across all income levels, with risks being more pronounced among those living alone compared to cohabiting mothers. These findings highlight how migration intersects with income and family structure, and can compound social disadvantage and elevate the risk of LBW. This categorization of migrants, which reflects global power inequalities, and its intersections with other social factors, represents a novel way of situating discussions about differences in low birthweight among immigrant mothers within the broader context of health inequalities. Moreover, our study contributes with a reflection on the theoretical limitations of standard analytical approaches based on ‘adjustments’ when addressing inequalities based on social stratification categories, as such approaches often ignore the variability that may exist within migrant categories. Our findings thus underscore the need for public health interventions that consider intersecting forms of social and economic disadvantage.

## Supplementary Information

Below is the link to the electronic supplementary material.

Supplementary Material 1

## Data Availability

These data cannot be made publicly available due to privacy regulations. According to the General Data Protection Regulation, the Swedish law SFS 2018:218, the Swedish Data Protection Act, the Swedish Ethical Review Act, and the Public Access to Information and Secrecy Act, these types of sensitive data can only be made available for specific purposes, including research, that meets the criteria for access to this type of sensitive and confidential data as determined by a legal review. Data are however available from the authors upon reasonable request after ethical approval and must be analysed at Stockholm University.
