# Supplementary material for: An intersectional approach to examine low birthweight by migration status in Sweden using register data
Source: Sci Rep. 2025 Nov 4;15:38464. doi: 10.1038/s41598-025-26172-0 (PMC12586708; doi:10.1038/s41598-025-26172-0)
Supplement: Supplementary file 1 — Supplementary Material 1 [file 41598_2025_26172_MOESM1_ESM.pdf]

Supplementary Table 1. Frequencies of low birthweight by combinations of region of birth, income, and partner support [1]. (n=1,917,171)

	**Swedish-born**		**Global North**		**Global South**	
	**n**	**n**	**n**	**n**	**n**	**n**
**Low Birthweight (< 2500 g)**	**No**	**Yes**	**No**	**Yes**	**No**	**Yes**
**Swedish/Cohabiting/High Income**	280,604	6,557				
**Swedish/Cohabiting/Medium Income**	387,745	8,545				
**Swedish/Cohabiting/Low Income**	303,360	7,143				
**Swedish/Alone/High Income**	274,595	9,704				
**Swedish/Alone/Medium Income**	149,032	5,238				
**Swedish/Alone/Low Income**	84,706	2,927				
**Global North/Cohabiting/High Income**			17,552	459		
**Global North/Cohabiting/Medium Income**			19,316	488		
**Global North/Cohabiting/Low Income**			26,947	724		
**Global North/Alone/High Income**			10,036	369		
**Global North/Alone/Medium Income**			5,704	220		
**Global North/Alone/Low Income**			9,266	343		
**Global South/Cohabiting/High Income**					22,924	841
**Global South/Cohabiting/Medium Income**					42,307	1,391
**Global South/Cohabiting/Low Income**					161,715	5,193
**Global South/Alone/High Income**					15,403	737
**Global South/Alone/Medium Income**					17,629	762
**Global South/Alone/Low Income**					35,359	1,330

[1] Partner support was proxied by information on household composition in the year prior to the child's birth, categorized as cohabitating or living alone.
